# Supplementary material for: Italian Survey on Endoscopic Biliary Drainage Approach in Patients with Surgically Altered Anatomy
Source: Medicina (Kaunas). 2024 Mar 13;60(3):472. doi: 10.3390/medicina60030472 (PMC10972116; doi:10.3390/medicina60030472)
Supplement: Supplementary file 1 [file medicina-60-00472-s001.zip › medicina-2888965-supplementary.pdf]

Endoscopic biliary drainage in altered anatomy: an Italian survey.

Section S1: expertise of endoscopic centers

How many ERCP/year are performed in your Center?
< 100 □
100-250 □
250-400 □
>400 □

How many EUS±FNA/FNB per year are performed in your Center?
< 250 □
250-500 □
500-750 □
>750 □

How many interventional EUS/year are performed in your Center?
< 10 □
10-20 □
20-50 □
>50 □

Is interventional radiology available in your Center?
Yes □
No □

Is biliopancreatic surgery available in your Center?
Yes □
No □

Is device assisted enteroscopy available in your Center?
Yes □
No □

Which is the rate of post-ERCP pancreatitis in your Center?
<5% □
5-10% □
>10% □
NA □

Which is the biliary cannulation rate in your Center?
<80% □
80-90% □
>90% □
NA□

Do you perform EUS-guided FNA/FNB?
Yes □

No □
If yes which is the rate of diagnostic tissue sampling ?

- <75 % □
- 75-85% □
- 85-90% □
- >90% □

Do you perform EUS guided drainage?
Yes □
No □
If yes which type of procedure?

- Drainage of pancreatic pseudocyst/walled-off necrosis □
- Drainage of gallbladder □
- Choledocoduodenostomy □
- Hepaticogastrostomy □
- Gastrojejunostomy □

Do you perform biliary drainage in patients with upper GI altered anatomy?
Yes □
No □
If Yes in which type of surgical reconstruction?

- Billroth II □
- Roux-en Y □
- Roux-en-Y gastric bypass □
- Post DCP/Whypple
- Others □ (specify)

Is your Center a referral for endoscopic biliary drainage in altered anatomy?
Yes □
No □

Do you refer patients for endoscopic biliary drainage in altered anatomy to other centers?
Yes □
No □

Do you refer patients for endoscopic biliary drainage in altered anatomy to other centers only if first attempt fails?
Yes □
No □

How many endoscopic biliary drainage/year are performed in your Center in patients with altered anatomy?
Number:

Section S2: Biliary drainage approach in case of Billroth-II reconstruction

Which is your first line endoscopic biliary drainage approach in patients with benign disease?

Conventional duodenoscope □
Device assisted enteroscopy □
Pediatric colonoscope or operative gastroscope or short enteroscope □
Laparoscopic assisted□
EUS- BD □
Percutaneous BD□
Surgical Rendez-vous□
Radiological Rendez-vous□
EUS-guided Rendez-vous□
Referral to other center □

And, in case of failure?
Conventional duodenoscope □
Device assisted enteroscopy □
Pediatric colonoscope or operative gastroscope or short enteroscope □
Laparoscopic assisted □
EUS- BD □
Percutaneous BD □
Surgical Rendez-vous□
Radiological Rendez-vous□
EUS-guided Rendez-vous□
Referral to other center □

Which is your first line endoscopic biliary drainage approach in patients with malignant disease?
Conventional duodenoscope □
Device assisted enteroscopy □
Pediatric colonoscope or operative gastroscope or short enteroscope □
Laparoscopic assisted□
EUS- BD □
Percutaneous BD □
Surgical Rendez-vous□
Radiological Rendez-vous□
EUS-guided Rendez-vous□
Referral to other center □

And, in case of failure?
Conventional duodenoscope □
Device assisted enteroscopy □
Pediatric colonoscope or operative gastroscope or short enteroscope □
Laparoscopic assisted □
EUS- BD □
Percutaneous BD □
Surgical Rendez-vous□
Radiological Rendez-vous□
EUS-guided Rendez-vous□
Referral to other center □

Section S3: Biliary drainage approach in case of Roux en Y reconstruction reconstruction:

Which is your first line endoscopic biliary drainage approach in patients with benign indication?
Device assisted enteroscopy □
Pediatric colonoscope or short enteroscope □
Laparoscopic assisted □
EUS- BD □
EUS-gastrojejunostomy assisted ERCP □
Percutaneous BD □
EUS-guided Rendez-vous □
Referral to other center □

And, in case of failure?
Device assisted enteroscopy □
Pediatric colonoscope or short enteroscope □
Laparoscopic assisted □
EUS- BD □
EUS-gastrojejunostomy assisted ERCP □
Percutaneous BD □
EUS-guided Rendez-vous □
Referral to other center □

Which is your first line endoscopic biliary drainage approach in patients with malignant indication?
Device assisted enteroscopy□
Pediatric colonoscope or short enteroscope□
Laparoscopic assisted□
EUS- BD□
EUS-gastrojejunostomy assisted ERCP□
Percutaneous BD□
EUS-guided Rendez-vous□
Referral to other center □

And, in case of failure?
Device assisted enteroscopy□
Pediatric colonoscope or short enteroscope □
Laparoscopic assisted□
EUS- BD□
EUS-gastrojejunostomy assisted ERCP□
Percutaneous BD□
EUS-guided Rendez-vous□
Referral to other center □
